# MicroRNA profiling of ovarian granulosa cell tumours reveals novel diagnostic and prognostic markers

**DOI:** 10.1186/s13148-017-0372-0

**Published:** 2017-07-21

**Authors:** Wei-Tzu Cheng, Roseanne Rosario, Anita Muthukaruppan, Michelle K Wilson, Kathryn Payne, Peter C. Fong, Andrew N. Shelling, Cherie Blenkiron

**Affiliations:** 1 Department of Obstetrics and Gynaecology, The University of Auckland, Auckland, New Zealand; 2 Centre for Reproductive Health, Queen’s Medical Research Institute, University of Edinburgh, Edinburgh, UK; 3 Department of Pathology, Auckland City Hospital, Auckland, New Zealand; 4 Department of Medical Oncology, Auckland City Hospital, Auckland, New Zealand; 5 Department of Molecular Medicine and Pathology, The University of Auckland, Private Bag 92019, Auckland Mail Centre, Auckland, 1142 New Zealand; 6 School of Biological Sciences, The University of Auckland, Auckland, New Zealand

**Keywords:** Tissue biomarker, Prognosticator, Ovarian cancer, Granulosa cell, Non-coding RNA

## Abstract

**Background:**

The aim of this study was to explore the clinical utility of microRNAs (miRNAs) as improved markers of ovarian granulosa cell tumours (GCTs) for cancer diagnosis and prognosis prediction. Current histopathological and genetic markers, such as the presence of a *FOXL2* gene mutation to distinguish between the two major subtypes are not wholly accurate and as such novel biomarkers are warranted.

**Methods:**

The miRNA expression profiles of five formalin-fixed, paraffin-embedded (FFPE) adult-GCTs and five juvenile-GCTs were assessed using Affymetrix miRNA 3.0 Arrays and compared for differential expression. Ten miRNAs were assessed in an additional 33 FFPE tumours and four normal granulosa cell samples by quantitative RT-PCR, and their expression correlated to clinical information.

**Results:**

MicroRNA array found 37 miRNAs as differentially expressed between the two GCT subtypes (*p* < 0.05, fold change ≥2 and among these, miRs -138-5p, -184, -204-5p, -29c-3p, -328-3p and -501-3p were validated by RT-qPCR as differentially expressed between the two GCT subtypes (*p* < 0.05). In addition, the expression of miR-184 was predictive of tumour recurrence in adult-GCTs, specifically for patients diagnosed with stage I and II and stage I only disease (*p* < 0.001 and *p* < 0.05, respectively).

**Conclusions:**

This study is the first to report on global miRNA expression profiles of human ovarian GCTs using FFPE tumour samples. We have validated six miRNAs as novel markers for subtype classification in GCTs with low levels of miR-138-5p correlating with early tumour stage. Low miR-184 abundance was correlated with tumour recurrence in early stage adult-GCT patients as a candidate predictive biomarker. Further studies are now needed to confirm the clinical utility of these miRNAs as diagnostic and recurrence markers, and understand their possible roles in the pathogenesis of GCTs.

**Electronic supplementary material:**

The online version of this article (doi:10.1186/s13148-017-0372-0) contains supplementary material, which is available to authorized users.

## Background

Granulosa cell tumours (GCTs) of the ovary are a unique subset of ovarian stromal tumours accounting for approximately 5% of all ovarian malignancies [1]. Based on their pathologic characteristics and clinical features, GCTs can be further divided into two distinct subtypes: adult-GCTs and juvenile-GCTs. Adult-GCTs are the predominant form (~95%) of GCTs and commonly present in women of early post-menopausal age. In contrast, juvenile-GCTs (~5%) typically occur in prepubertal girls and young women. In general, GCT patients often manifest endocrine symptoms that are associated with oestrogen hypersecretion by the tumour, including abnormal uterine bleeding, precocious puberty, cyclic irregularities and menorrhagia, thus allowing the tumour to be detected and diagnosed at an earlier stage according to the International Federation of Gynaecology and Obstetrics (FIGO) staging system [2–4]. Consequently, the majority of GCT cases are diagnosed in stage I and these patients typically have a favourable prognosis with a >90% overall 5-year survival rate [5]. However, the high propensity of late disease relapse means the onset of recurrence is unpredictable, as GCTs are known to recur many years following the initial diagnosis, making surveillance difficult to conduct. Prolonged postoperative follow-up has therefore been suggested to be mandatory in the disease management of GCT patients [6].

GCTs are believed to arise from granulosa cells that surround the oocyte in the developing follicle [1]. However, despite their common cellular origin, the two subtypes appear to exhibit very distinct histological and clinical characteristics. Histologically, adult-GCT is characterised by a variety of growth patterns, often admixed, including diffuse, trabecular, insular and microfollicular (including unique microcystic spaces known as Call-Exner bodies), and the presence of distinct nuclear grooving in tumour cells [7], whereas juvenile-GCTs appear as diffuse follicular cysts with no nuclear grooves [8]. Whilst GCTs are generally associated with a good clinical outcome, up to 80% of adult-GCT patients with recurrence may eventually succumb to the disease [9]. In contrast, tumour relapse rarely occurs in juvenile-GCT patients [10, 11]. This difference between adult- and juvenile-GCTs highlights the importance of accurate subtype distinction at clinical diagnosis, as misclassification of GCT subtypes may result in suboptimal recurrence surveillance and patient follow-up. Currently, the distinction between subtypes is exclusively based on histological assessment of tumour morphology; however, the subtle distinctions in their histopathological presentation present several technical challenges in accurate subtype classification of GCTs [12, 13].

The search for clinically useful biomarkers for GCTs has been greatly impeded, in part, by its rarity. The recent identification of a single, recurring somatic mutation (c.402 C>G; p.C134W) in the *FOXL2* gene almost exclusively in adult-GCTs has suggested the possibility of implementing *FOXL2* gene mutation analyses into current diagnostic practice [12]. Subsequent studies not only independently validated the specificity of the *FOXL2* mutation for adult-GCTs, but also confirmed its absence in juvenile-GCTs and in other unrelated human tumours [12–19]. Although the high specificity of the *FOXL2* mutation for adult-GCTs makes it an attractive molecular marker for subtype classification, its diagnostic power has been challenged by reported cases of mutation-negative adult-GCTs and also cases of mutation-positive juvenile-GCTs [12, 13] [15, 16, 18, 20]. Consequently, the lack of substantial evidence of the *FOXL2* mutation as a definitive diagnostic marker of GCTs warrants the need for a more specific and robust diagnostic marker. In this study, we investigate the use of microRNAs (miRNAs) as potential biomarkers of GCTs.

MicroRNAs are endogenously expressed, short non-coding RNAs, which post-transcriptionally repress the expression of their gene targets in order to indirectly regulate biological functions. miRNAs act as ‘gene silencers’ by binding to complementary sequences, usually present in the 3′ untranslated region (UTR) of messenger RNA transcripts, targeting these transcripts for enzymatic degradation or translational inhibition [21]. MiRNAs have demonstrated several important diagnostic and prognostic implications in clinical studies [22, 23]. Whilst the miRNA expression of human GCT-derived cell lines that are representative of the two clinical subtypes have been profiled previously [24], no studies to date have investigated miRNA expression differences of the two subtypes using human tumour samples.

In this study, we conducted miRNA expression profiling of adult- and juvenile-GCTs using paraffin-embedded tumours. Using this information, we aimed to identify miRNAs that are differentially expressed by the two subtypes and thus may serve as potential novel molecular markers for clinical diagnosis and subtype classification. Furthermore, we investigated the prognostic power of miRNAs in predicting tumour recurrence in GCT patients.

## Methods

### Case selection

Adult-GCTs and juvenile-GCTs included in this study were retrospectively selected from cases of ovarian GCTs registered in the Auckland Regional Gynaecology Multidisciplinary Team database, Auckland, New Zealand (NZ), and the Cancer Society Tissue Bank, Christchurch, NZ, between 1955 and 2011, with ethical approvals from the Multi-Region Ethics Committee, Ministry of Health, NZ (MEC.09.10.111). In total, formalin-fixed, paraffin-embedded (FFPE) tumour blocks were available for 37 cases of adult-GCTs and 6 cases of the rarer juvenile-GCTs. Information on the clinical history and pathologic findings were documented until 31 December 2013. FFPE tumours J1-J5 and A1-A5 were included in the test array set whilst J6 and the remaining 32 adult-GCT samples(A6-A37) were used in the additional validation set (Additional File 1). The *FOXL2* gene mutation status of the six juvenile-GCT samples was analysed using methods described previously [16]. The clinico-pathological characteristics of the 37 adult-GCT cases, including the *FOXL2* gene mutation status, have been described previously [16].

### MicroRNA expression profiling by microarray

Total RNA was extracted from paraffin-embedded samples using the RecoverAll Total Nucleic Acid Isolation Kit for FFPE (Ambion, NZ) following the manufacturer’s protocols. Microarray analysis using Affymetrix GeneChip miRNA 3.0 Arrays (Affymetrix, CA, USA) was performed using methods described previously [24]. The microarray dataset has been deposited in NCBI’s Gene Expression Omnibus (GEO) and is accessible through GEO Series accession number GSE70026 (http://www.ncbi.nlm.nih.gov/geo/query/acc.cgi?token=gdubicuafjulviz&acc=GSE70026). Differential expression analysis was performed using one-way ANOVA and miRNA probe sets with a *p* value threshold of <0.05, a fold change ≥2 or ≤−2 and a false discovery rate (FDR) ≤0.2 were considered to be differentially expressed.

### Cell culture

Human granulosa cells were obtained from patients undergoing in vitro fertilisation treatment at the Fertility Associates clinic, Auckland, NZ, with approvals from The University of Auckland Human Participants Ethics Committee, The University of Auckland, NZ (reference 011386). Granulosa cells were snap-frozen following oocyte collection, and total RNA was extracted using RNAqueous-Micro Total RNA Isolation Kit (Ambion) following the manufacturer’s protocols.

### MicroRNA expression profiling by quantitative RT-PCR

The expression of ten selected miRNAs was assessed in the original cohort plus additional GCT tumour samples using quantitative RT-PCR (RT-qPCR; 32 adult, 1 juvenile extra). A subset of seven miRNAs was selected based on the array findings. In addition, miRs -138-5p, -21-5p and -29c-3p were included in the validation as these have been previously implicated in the biology of GCTs and other types of ovarian cancer [24–26]. Total RNA from normal granulosa cells and GCT samples was reverse transcribed using the TaqMan MicroRNA Reverse Transcription Kit (Applied Biosystems) and pre-amplified using methods modified from the manufacturer’s protocols. Quantification of miRNA candidates was performed in triplicate using predesigned TaqMan MicroRNA Assays on the QuantStudio 12K Flex Real-Time PCR System (Applied Biosystems). Relative miRNA abundance was calculated using the comparative C_T_ method using RNA U6 as an endogenous control [27]. Statistical significance of differences in miRNA abundance between clinical groups was tested by the Wilcoxon-Mann-Whitney Rank Sum test using the PRISM 6.01 GraphPad software programme (California, USA). Recurrence-free survival analysis was performed using the Log-Rank test using the ‘survival’ package in R and the Kaplan-Meier estimates of survival probability in the PRISM 6.01 GraphPad. Correlations between miRNA RT-qPCR data and clinico-pathological features were assessed using a two-tailed unequal variance test.

## Results

### Patient characteristics and FOXL2 gene mutational status

The clinical and pathological characteristics of the six juvenile-GCT patients (and all adult-GCTs) are reported in Additional file 1. The median age at diagnosis was 11 years (range 8–27). The majority were diagnosed with FIGO stage I tumours (5/6), although one patient had FIGO stage II disease at presentation. The median follow-up time following diagnosis was 6.2 years (range 2.8–8.7 years) and with no recurrences seen during this time, as is common for this subtype. The *FOXL2* gene mutation status of the six juvenile-GCT tumours was determined by direct DNA sequencing. Five (J1-J5) out of six patients carried the wildtype *FOXL2* allele, further confirming the initial clinical diagnosis of these tumours (Additional file 1). Surprisingly, one juvenile-GCT sample (J6) had the c.402 C>T *FOXL2* mutation.

### Differential miRNA expression profiles between adult- and juvenile-GCTs

**Fig. 1:**
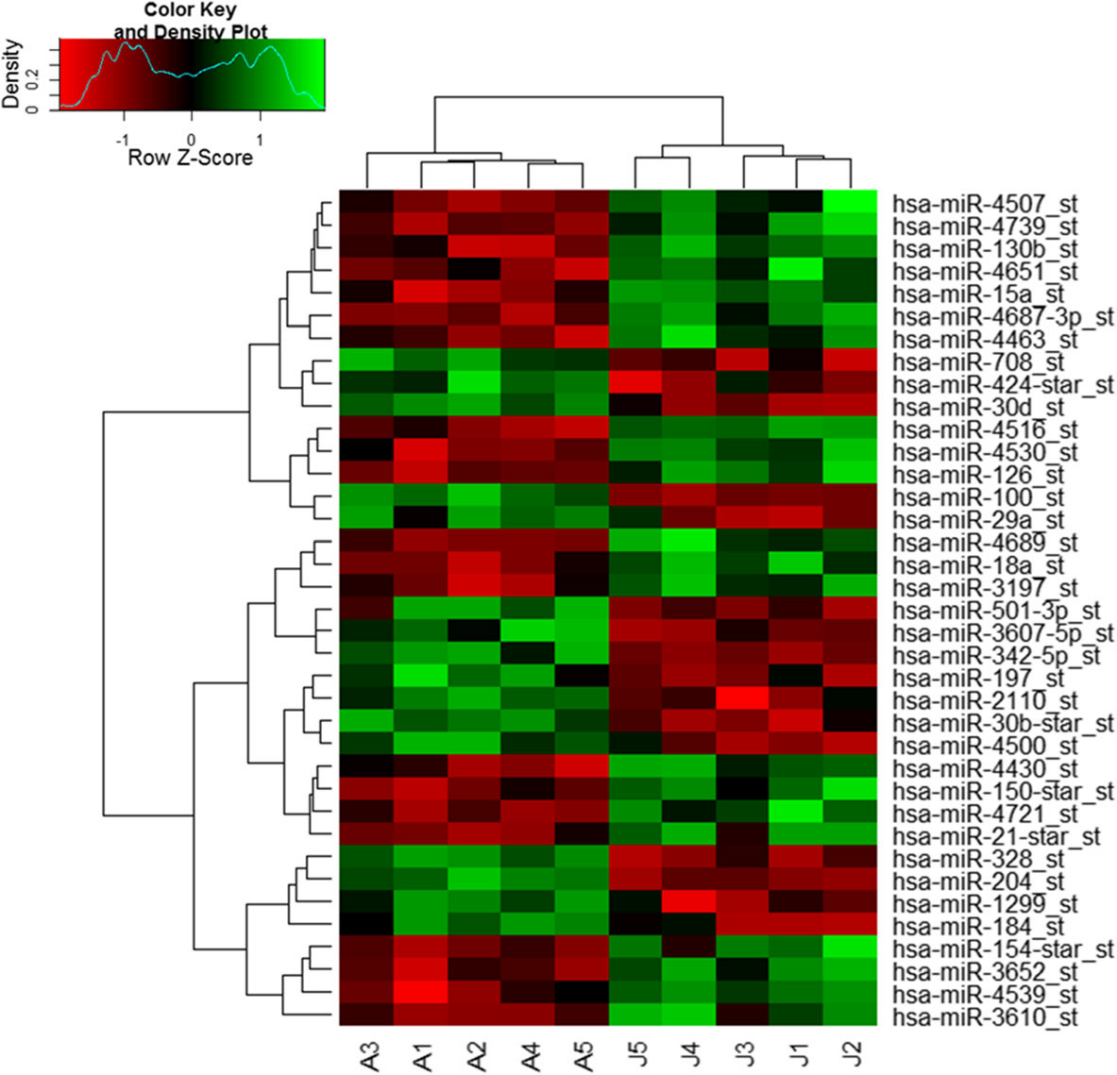
Differentially expressed miRNAs between adult- and juvenile-GCTs as assessed by miRNA expression microarrays. The differential miRNA expression profiles of five adult (labelled as A1-A5) and five juvenile (labelled as J1-J5) GCTs (test array set) are hierarchically clustered based on the relative abundance of 37 differentially expressed miRNA probe sets between the two subtypes (ANOVA, *p* < 0.05, fold change ≥2 or ≤−2, FDR ≤0.2). *Red* and *green* represent low and high relative abundance of the indicated miRNA, respectively

We compared the miRNA expression profiles of the adult- and juvenile-GCT samples of the test array set using ANOVA and identified miRNA expression signatures that were unique to the two GCT subtypes (Fig. 1). In total, 37 miRNAs were found to be significantly differentially expressed between adult- and juvenile-GCTs. Of those, 16 miRNAs were more abundant in adult-GCTs and conversely 21 miRNAs were more abundant in juvenile-GCTs (Table 1 and Table 2, respectively). Interestingly, among the differentially regulated miRNAs, miR-184 was shown to be 56 folds more highly abundant in adult-GCTs when compared to juvenile-GCTs (Table 1). A permutation analysis was performed and demonstrated that the miRNA array data was robust despite the small sample size (Additional file 2).

**Table 1:** List of 16 miRNA probe sets more abundant by >2 fold in the adult-GCT subtype as determined by miRNA expression microarrays

Transcript ID	miRBase identifier	Fold change (vs. juvenile)^a^	ANOVA *p* value	FDR *p* value
*hsa-miR-184*	MIMAT0000454	56.04	0.004	0.171
*hsa-miR-204-5p*	MIMAT0000265	14.48	<0.0001	0.011
*hsa-miR-328-3p*	MIMAT0000752	11.87	<0.0001	0.032
hsa-miR-1299	MIMAT0005887	8.58	0.003	0.163
hsa-miR-342-5p	MIMAT0004694	3.78	<0.0001	0.033
*hsa-miR-501-3p*	MIMAT0004774	3.65	0.002	0.148
hsa-miR-708-5p	MIMAT0004926	3.41	0.001	0.111
hsa-miR-100-5p	MIMAT0000098	2.98	<0.0001	0.011
hsa-miR-3607-5p	MIMAT0017984	2.96	0.001	0.120
hsa-miR-29a-3p	MIMAT0000086	2.84	0.003	0.151
hsa-miR-197-3p	MIMAT0000227	2.71	0.003	0.156
hsa-miR-30d-5p	MIMAT0000245	2.58	0.0001	0.045
hsa-miR-2110	MIMAT0010133	2.57	0.002	0.148
hsa-miR-4500	MIMAT0019036	2.45	0.001	0.119
hsa-miR-30b-star (-3p)	MIMAT0004589	2.34	0.0003	0.058
hsa-miR-424-star (-3p)	MIMAT0004749	2.11	0.005	0.193

miRNAs in italics were tested in a further validation set of GCTs

*Transcript ID* transcript identifier, *FDR* false discovery rate

^a^Fold change of miRNAs was expressed relative to the juvenile subtype

**Table 2:** List of 21 miRNA probe sets more abundant by >2 fold in the juvenile-GCT subtype as determined by miRNA expression microarrays

Transcript ID	miRBase identifier	Fold change (vs. adult)^a^	ANOVA *p* value	FDR *p* value
*hsa-miR-21-star (-3p)*	MIMAT0004494	8.63	0.001	0.114
hsa-miR-154-star (-3p)	MIMAT0000453	5.24	0.001	0.120
*hsa-miR-15a-5p*	MIMAT0000068	5.11	0.0006	0.073
hsa-miR-3610	MIMAT0017987	3.70	0.001	0.118
hsa-miR-4689	MIMAT0019778	3.29	0.0005	0.069
hsa-miR-130b-3p	MIMAT0000691	3.17	0.0007	0.077
hsa-miR-4430	MIMAT0018945	2.97	0.001	0.107
hsa-miR-4721	MIMAT0019835	2.89	0.0009	0.091
hsa-miR-4687-3p	MIMAT0019775	2.74	0.0001	0.039
hsa-miR-4507	MIMAT0019044	2.64	0.002	0.140
hsa-miR-4530	MIMAT0019069	2.58	0.0009	0.089
hsa-miR-18a-5p	MIMAT0000072	2.52	0.001	0.092
hsa-miR-150-star (-3p)	MIMAT0004610	2.46	0.001	0.105
hsa-miR-4463	MIMAT0018987	2.27	0.001	0.109
hsa-miR-3652	MIMAT0018072	2.22	0.0007	0.080
hsa-miR-4516	MIMAT0019053	2.19	0.0001	0.039
hsa-miR-4651	MIMAT0019715	2.18	0.002	0.128
hsa-miR-4739	MIMAT0019868	2.13	0.001	0.093
hsa-miR-4539	MIMAT0019082	2.11	0.001	0.115
hsa-miR-126-3p	MIMAT0000445	2.03	0.0003	0.058
hsa-miR-3197	MIMAT0015082	2.03	0.003	0.151

miRNAs in italics were tested in a further validation set of GCTs

*Transcript ID* transcript identifier, *FDR* false discovery rate

^a^Fold change of miRNAs was expressed relative to the adult subtype

### miRNA expression profiles of GCTs and granulosa cells

**Fig. 2:**
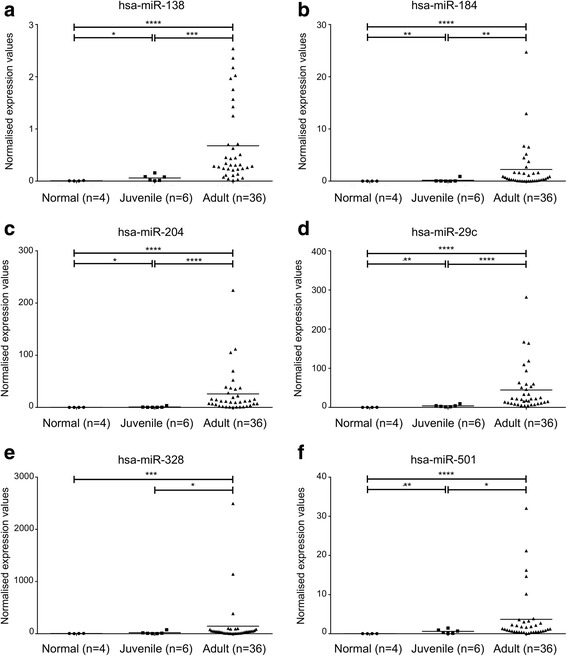
Quantitative RT-PCR validation of microRNA expression in GCTs. Normalised expression values of **a.** hsa-miR-138-5p, **b.** hsa-miR-184, **c.** hsa-miR-204-5p, **d.** hsa-miR-29c-3p, **e.** hsa-miR-328-3p, and **f.** hsa-miR-501-3p in healthy normal granulosa cells (*circle*), juvenile-GCT (*square*) and adult-GCT (*triangle*) assessed using RT-qPCR. The points depict the normalised expression value (relative to RNU6B) for individual samples. The *horizontal bars* represent the mean expression value for each group. (*) indicates the level of statistical significance as determined by a two-tailed, Wilcoxon-Mann-Whitney Rank Sum test where * *p* < 0.05, ** *p* < 0.01, *** *p* < 0.001 and **** *p* < 0.0001

**Fig. 3:**
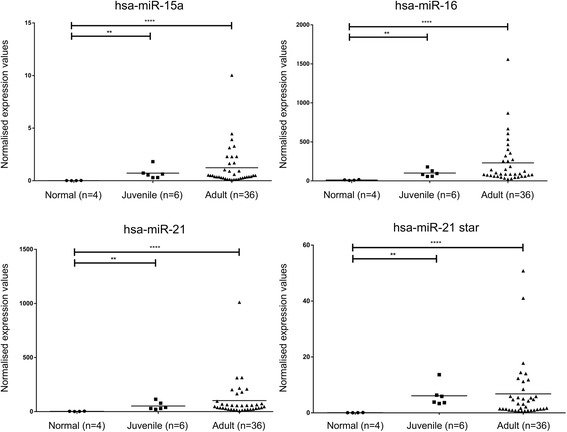
Normalised expression values of hsa-miR-15a in adult-GCT, juvenile-GCT and healthy normal granulosa cells. The *horizontal bar* represents the mean expression value for each group. * indicates the level of statistical significance as determined using a two-tailed, Mann-Whitney rank sum test where ** and **** represent *p* < 0.01 and *p* < 0.0001, respectively

The expression profiles of ten selected miRNAs were further assessed in the additional validation set using RT-qPCR. Healthy human granulosa cells were also included to assess relatively deregulated miRNA expression in tumours. Comparisons with the granulosa cell controls revealed significant differences in patterns of miRNA expressions between the two GCT subtypes (Fig. 2). Whilst adult-GCTs appeared to have a high abundance of most miRNAs, the lower miRNA expression profile of juvenile-GCTs more closely resembled that of normal granulosa cells. miRs -138-5p, -184, -204-5p, -29c-3p, -328-3p and -501-3p were significantly more abundant in adult-GCTs when compared to juvenile-GCTs (*p* < 0.05) and normal granulosa cell control (*p* < 0.05) (Fig. 2a–f). In contrast, four of the ten miRNAs chosen for RT-qPCR gene expression analysis (miRs -15a-5p, -16-5p, -21-5p and -21-3p) were not different between the two subtypes in this GCT cohort, although the abundances were significantly higher in GCTs of either subtype when compared to normal granulosa cell controls (*p* < 0.05) (Fig. 3).

### Predicted pathways and mRNA targets of miRNAs abundant in adult-GCTs

We used miRPath v.3 [28] to identify any signalling pathways that miRs -138-5p, -184, -204-5p, -29c-3p, -328-3p and -501-3p converged onto that might be relevant in the pathogenesis of adult-GCTs, as these miRNAs were significantly more abundant in this tumour subtype compared to juvenile-GCTs. In this analysis using microT-CDS in silico target predictions, at least five miRNAs had predicted mRNA targets (genes union) involved in KEGG pathways ECM-receptor interaction (hsa04512; *p* < 0.0001; 5 miRNAs; 24 genes), PI3K-Akt signalling pathway (hsa04151; *p* < 0.0001; 6 miRNAs; 65 genes), and focal adhesion (hsa04510; *p* < 0.0001; 5 miRNAs; 46 genes). Experimentally supported TarBase targets for all miRNAs but miR-328-3p, which was not present in the database, also confirmed these as significantly converging pathways. BCL2, involved in apoptosis (hsa04210) and PI3K-Akt signalling (hsa04151), was a common TarBase target for four of the six miRNAs (miR-138-5p, -204-5p, -29c-5p and -501-3p).

### Identification of predictive miRNA markers for GCT recurrence

**Fig. 4:**
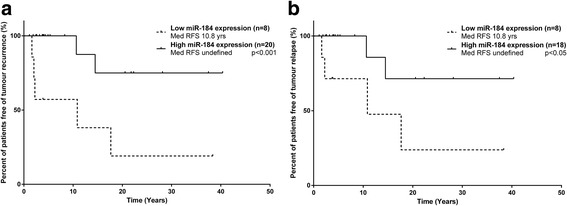
MiRNA-184 expression correlates with GCT patient survival. Kaplan-Meier estimates of recurrence-free survival for **a.** stage I and II and **b.** stage I only adult-GCT patients according miR-184 expression. 30th quantiles was used as the expression cut-off for miR-184. Med RFS: Median recurrence-free survival

Irrespective of their predicted biological function, the miRNAs identified could be ideal molecular markers not only for diagnosis of GCT subtypes but also for relapse prediction. Specifically, we focused on adult-GCT patients as this is the clinical subtype that is often characterised by frequent and late tumour recurrence. We note that none of the juvenile-GCT patients developed tumour recurrence. Tumour recurrence information on 34 adult-GCT patients was available: 26 stage I (76%) patients, two stage II (6%) patients and six stage III (18%) patients (Additional file 1). Of those, 13 (38%) patients including all six stage III patients recurred following the initial diagnosis and during the follow-up of the study. As tumour stage is a known independent prognostic factor for GCTs, we limited our analysis to only stage I and II cases to test our miRNAs for their ability to stratify these patients by recurrence. Patients were grouped into ‘stage I and II’ (*n* = 28) and ‘stage I only’ (*n* = 26) and then divided into either high or low miRNA expression groups. Recurrence-free survival was compared between patients of higher and lower expression groups using a log-rank test. We found a significant difference in recurrence-free survival between tumours with low expression of miR-184 when compared to those with high expression for both ‘stage I and II’ and ‘stage I only’ patients (*p* < 0.05) (Fig. 4). Patients with a low tumour miR-184 expression had a significantly shorter median time to disease recurrence when compared to those with high tumour expression. Statistical analysis of other miRNAs relative to recurrence and other clinico-pathological variables (listed in Additional file 1) revealed only miR-138 as having a significantly higher expression in lower Stage I tumours (All GCTs *p* = 0.012; Adult-GCTs only *p* = 0.009; >stage I mean 0.29, <stage I mean = 0.71).

## Discussion

In this investigation, we have broadened our search for GCT-specific molecular markers from *FOXL2* (c.402 C>G) to include miRNAs, an increasingly important class of tissue biomarkers that has been widely investigated in several other human malignancies [22]. Using a small cohort of ten archival GCT tissues (test array set), we were able to identify 37 miRNAs that were demonstrated to be differentially regulated by the two histological GCT subtypes. The expression differences for ten differentially regulated miRNAs were validated in a larger cohort (validation set of 33 GCTs) which confirmed that the two GCT subtypes have distinct miRNA signatures and showed that the juvenile-GCTs resemble normal granulosa cells more than adult-GCTs. The array data identified hsa-miR-184 to be 56 times more abundant in adult-GCTs than juvenile-GCTs, a difference later validated by RT-qPCR.

miR-184 is highly conserved across animal kingdoms and its expression has been shown to be critical in regulating early developmental processes in *Drosophila melanogaster*, particularly in female germline development as well as oogenesis [29]. miR-184 has also been shown to be expressed in normal proliferating granulosa cells of the developing follicle in bovine animals [30]. During follicular development, miR-184 regulates steroidogenesis and follicle maturation, two important biological functions of normal granulosa cells [30]. Therefore the abundant expression of miR-184 in the adult-GCT tumours is consistent with its known cellular origin and suggest that miR-184 is likely to have a role in normal granulosa cell biology which, we believe, becomes perturbed in GCTs. In addition to the unique GCT-specific *FOXL2* mutation, it appears that abundant miR-184 is also a characteristic molecular signature of adult-GCTs.

miR-184 in human cancer appears to be context-dependent with roles as an oncogene and a tumour suppressor gene [31–36]. Considering its involvement in various types of human cancer, the expression of miR-184 as a tumour biomarker has been extensively investigated. In prostate cancer, for example, miR-184 is differentially expressed in cancer cells of higher-grade tumours with a more aggressive clinical behaviour when compared with lower-grade tumours [37]. Contrastingly, our study has demonstrated that early FIGO-staged adult-GCT tumours expressing low tumour miR-184 expression at disease presentation were significantly more likely to recur when compared to tumours with high expression. The inverse correlation between miR-184 expression and disease recurrence would suggest to us that miR-184 is probably tumour suppressive in the context of GCTs, which is supported by its expression patterns seen in other tumour types [34–36]. However, as we were limited by the study sample size, validation of this finding in a larger sample series is warranted in order to confirm and fully elucidate the predictive power of miR-184 as a recurrence marker.

The reduction of miR-184 in earlier recurring tumours may also be biologically relevant. Tarbase v7.0 [38] listed *LAMC1* as an experimentally confirmed and in silico predicted target of miR-184. LAMC1, laminin subunit gamma 1, an extracellular matrix structural glycoprotein, is inherently associated with granulosa cell function increasing during follicular development [39], with gene variants linked to premature ovarian failure [40], a disease that is also associated with errors in *FOXL2*, and the gene also mutated in adult-GCT. Other published targets, linked to an involvement in the regulation of apoptosis and tumour invasion, include BCL2 [41], CDC25A [42] and TNFAIP2 [43]. Further, miR-184 has been linked to regulation of the PI3K/AKT/mTOR pathway [44], via multiple confirmed gene targets, which is a key signalling pathway in the development of GCTs [45, 46]. We would propose that loss of miR-184 in the tumours may in part lead to earlier recurrence due to the loss of inhibition of migration and invasion promoting genes [47].

A set of six miRNAs were validated as having a higher abundance in adult-GCTs than in juvenile-GCTs or normal granulosa cells (Fig. 2) and therefore could be specific molecular markers of GCT subtype. These six miRNAs were predicted to have converging regulation of mRNAs involved in ECM-receptor interaction, focal adhesion and the PI3K/Akt signalling pathways, with four miRNAs specifically targeting apoptosis regulator *BCL2*. High BCL2 expression in GCTs has been shown to correlate with a low proliferation rate as measured by ki-67 and mitotic index and with tumours smaller <10 cm, therefore with low-malignancy GCTs [48]. The six miRNAs did not correlate with these features although a trend towards significance (*p* < 0.09) was seen for miR-138 and miR-204 in the <10-cm tumours.

Interestingly, all of the ten miRNAs assessed by RT-qPCR exhibited their highest expression in the adult-GCT group, even when they were not significantly different from the juvenile-GCTs or normal granulosa cells (Figs. 2 and 3). We note that the RNU6B housekeeping gene was invariant between groups (*p* > 0.05). This general increase in miRNA abundance in the adult-GCTs hints towards a more active global miRNA expression or processing pathway, a phenomenon that has also been observed in several other human malignancies [49–51]. A global increase in miRNA biogenesis and activity in human cancer is often linked to a global suppression of miRNA target genes, especially those that are tumour suppressive in nature, thereby promoting malignant behaviour of cancer cells.

In addition to miRNA profiling, we also analysed the *FOXL2* mutation status of the juvenile-GCT tumours (Additional file 1) and demonstrated that the majority of the juvenile-GCT tumours (5/6) carried the wildtype *FOXL2* allele, a finding that is consistent with the high specificity of *FOXL2* mutation for adult-GCTs [12–15, 17–20]. However, one juvenile-GCT tumour (J6) was shown to harbour the *FOXL2* mutation. This unexpected observation is similar to the report of one *FOXL2* mutation-positive tumour out of ten juvenile-GCT tumours evaluated by Shah et al. [12]. Upon histological reassessment by two independent gynaecological pathologists, the morphology/cytology of this juvenile-GCT tumour was described to be unusual with a solid growth pattern and presence of necrosis and inflammation. The nuclei of tumour cells appeared to be oval shaped. Moreover, on closer examination, distinct coffee bean-like nuclear grooving, a distinctive feature of adult-GCTs, was present in some nuclei. In contrast, Call-Exner bodies, another microscopic feature that is considered diagnostic of adult-GCTs [7], and other sex cord components were not observed. A histological diagnosis of adult-GCT was concluded upon pathological review. Interestingly, this particular tumour also had the highest level of miR-184 expression of all the juvenile-GCT tumours included in this study (Fig. 2b), which further supports that it was an adult-GCT that might have been misdiagnosed based on standard pathology diagnostics. This sample also highlights the importance for finding new molecular markers and combining these with *FOXL2* mutation screens and standard morphological review for correct adult-GCT diagnosis.

## Conclusions

Currently, there is a clinical need for a more comprehensive, robust molecular marker for the diagnosis and tumour subtyping of GCTs. To the best of our knowledge, this is the first detailed study on the global miRNA expression profiles of human ovarian GCTs using paraffin samples. We have established that adult-GCTs and juvenile-GCTs have significantly different miRNA expression profiles and should therefore be considered as two biologically distinct tumours. In particular, miRs -138-5p, -184, -204-5p, -29c-3p, -328-3p and -501-3p were demonstrated to be differentially regulated by the two GCT subtypes. Therefore, we propose the possibility of incorporating miRNA expression signature profiling at the time of diagnosis for tumour subtyping, as well as using miRNA expression to risk-stratify GCT patients for prioritisation of patient follow-up. This study has demonstrated the clinical potential of miRNAs in the context of ovarian GCTs, and should now be validated in further cohorts and future prospective studies.

## Additional files

Additional file 1: Clinico-pathological characteristics of GCT tumours included in this study. *Adult-GCT patient samples with unknown FIGO stage information and excluded from recurrence analysis; †Adult-GCT patient sample with an aggressive clinical progression (progressive disease and tumour recurrence) and excluded from recurrence analysis; ^a^Clinical diagnosis based on histopathology**;** ^b^Staged according to the International Federation of Gynaecology and Obstetrics (FIGO) system. (XLSX 13 kb)

Additional file 2: Density distribution of *p* values between adult-GCT and juvenile-GCT tumours in the miRNA microarray dataset. The red and grey lines represent the *p* value density distributions for the true GCT groups in the miRNA dataset and for the 100 permuted and resampled groups, respectively. (TIFF 48 kb)

## Abbreviations

FDR: False discovery rate
FFPE: Formalin fixed paraffin embedded
FIGO: International Federation of Gynaecology and Obstetrics
GCT: Granulosa cell tumour
GEO: Gene Expression Omnibus
miRNA: MicroRNA
NZ: New Zealand
UTR: Untranslated region
