# Supplementary figures and images for: MicroRNA profiling of ovarian granulosa cell tumours reveals novel diagnostic and prognostic markers
Source: Clin Epigenetics. 2017 Jul 21;9:72. doi: 10.1186/s13148-017-0372-0 (PMC5521084; doi:10.1186/s13148-017-0372-0)

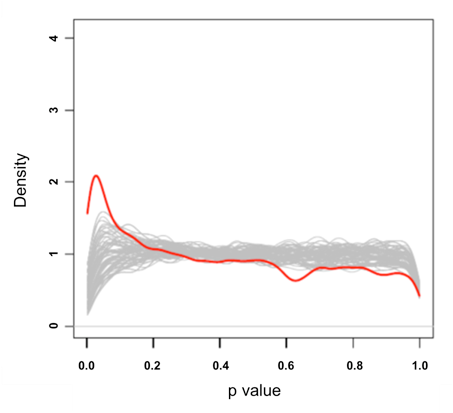

Supplement: Supplementary file 2 — Density distribution of p values between adult-GCT and juvenile-GCT tumours in the miRNA microarray dataset. The red and grey lines represent the p value density distributions for the true GCT groups in the miRNA dataset and for the 100 permuted and resampled groups, respectively. (TIFF 48 kb) [file 13148_2017_372_MOESM2_ESM.tif]
